# To Junior Dentists

**Published:** 1885-10

**Authors:** 


### Editorial.

### TO JUNIOR DENTISTS.

(It is a relief occasionally to lay aside the formal and absurdly ponderous editorial "we," and to speak as if face to face with the reader. One who is engaged in practice, and who at the same time has his regular modicum of "copy" to grind out for the printers, cannot wait for inspirations, but must humor his moods, and write, like Burns, as—

"———time and chance determine;
Perhaps it may turn out a sang, perhaps turn out a sermon."

Will the readers of this journal, then, kindly indulge a little whimsicality and capriciousness in these pages, and accept the song in hope of the sermon which they may get at another time?)

I am not an old man. I indignantly denounce the miscreant who dares to insinuate such an accusation. But then I am not a young man either—worse luck. Perhaps I had better compromise with Time, and admit that I am just at the summit of existence. But that implies that henceforth my path lies down the hill of life. There may possibly be a long road ahead for me to travel, but—it is a long road that I have come over. I may have years to look forward to, but it is certain that a good many years lie behind me. What the future may yield I know not, but all these past professional years have been years of toil, of the very hardest of hard work, of fighting against discouragements in the endeavor to make a place in the world, though it has not, I am thankful to say, been *all* discouragement. There has been a wonderful deal of enjoyment connected with the past, of pleasure in my work, and of honest, soulful exultation when I felt that I had scored a point in the game of existence. Take it for all in all, my professional life has not been an unhappy one. Every sorrow has had its compensating joy, and there have been many days of rejoicing scarcely alloyed by a regretful recollection or pang. Mine has been a busy life, and for that I am devoutly thankful, for I am fully satisfied that it is only the man whose time is occupied by some absorbing and congenial occupation who is really contented and happy. Therefore, I say I am grateful that mine has been a busy life.

Why do I start off in such a vein as this? Perhaps it is because of an association of ideas. I had occasion not long since to write an article that was retrospective in its nature, and it may be that I pick up the pen where I laid it down. Possibly my present mood takes its tone from the character of certain letters which I have lately received. My correspondence is considerable, and I find that of late years I am frequently appealed to by young dentists who ask my advice in all sorts of emergencies, as of an experienced practitioner. They seem to be rather assuming the tone of students to their tutor, or of juniors to their senior, and this causes me to enquire if I am really becoming a senior in a profession where I have so long felt myself a junior, and to rather regret the years that are passed. I have been in practice—let me see—it is now more than t-w-e-n-t-y y-e-a-r-s. Really, I *am* getting to be a senior. Well, if I must perforce be so disagreeably reminded of the flight of time, I will assume the airs of the position and demand its privileges, one of which is that of lecturing the young—of patronizing and pedagoging them.

A favorite amusement for the idle mind is to write me for my opinion on all sorts of topics. For instance, a few of the letters which I have lately received ask information as follows: Concerning the working of the Dental Law in the State of New York; advice about the inauguration of a microscopical society; how I would treat alveolar abscess; concerning the habits of the American Crow (much I know about Crow); the histological structure and probable character of a tumor, a bit of which was enclosed (nothing concerning the history of the case was sent); what is my cure for toothache; what kind of gold I would recommend for a young dentist to employ, with instructions in its use so that he can make a first-class filling.

All these enquiries are *bona fide*, and come from those who are really anxious to learn. They appeal to me as one who has had experience, and their applications cannot therefore be disregarded. Some enquiries are rather crude, and some possibly impertinent, but they are not so intended, and I take them in the spirit in which they are made and answer as best I may, remembering the time when I, too, was groping in the dark, and endeavoring to get hold of the right end of subjects which have since become more familiar to me. Perhaps if to some of these letters I set a decent text, and preach through the columns of the INDEPENDENT PRACTITIONER, I may get a larger audience than I can secure for private letters, and possibly, too, I may be enabled to lubricate some of those exceedingly rough places where I can remember sticking in days that are gone by. There is a constant influx of young dentists into the profession; all of them have things to learn which the colleges do not teach them, and which can only be acquired by stern personal experience, or through the relation of that of others. The former way is a sure and certain one, but it is slow, and awfully expensive. Possibly the long years spent by older practitioners in climbing over certain obstacles may, if their history be given, teach the younger ones a shorter way around. The writers of the letters of which I am in frequent receipt are but types of others, therefore let my experience speak to the class, and not to a single individual.

A. writes to know what gold and what instruments he shall use to produce first-class work. My dear boy, you have the hardest lesson in life yet to learn, and that is that there is no patent on ability; that there is no short cut to excellence, and that first-class dentistry is the result of industry, and not of special methods, materials and instruments. The boy working beside the experienced man is always wanting to trade tools with him, but when the exchange is made he finds himself no better off. He thinks his special job is an unusually difficult one, but when he obtains the place of the other he finds he has gained nothing. He anathematizes his material, and thinks if he were possessed of some like that of the man whose work seems so easy, he would then be happy, but this done, his task is no easier. It is the *skill* which he lacks, and that skill is the result of long years of patient toil and practice. A good operator will work almost any foil. The tyro will find extreme difficulty in using the best. A few general principles will, perhaps, be of assistance to you, but they will not, unless you have the industry to patiently work them out to a successful termination.

The softer and less adhesive the gold, the better will be its adaptation to the walls of the cavity, because the separate layers will not cohere and bridge over minute inequalities, thus leaving empty chasms in the peripheral walls, but will slide the one upon the other until they reach absolute contact with the tooth substance. Examine some of the soft gold fillings inserted by the old-time operators of forty years ago, before the date of cohesive foil, and which not infrequently will have been found to remain in place for all that length of time, retained only by being wedged in the cavity, and you will comprehend what is meant by perfect adaptation. Such fillings will be found to have preserved the teeth perfectly, but they have no such thing as contour. That belongs to a later age, and is produced by a totally different kind of foil. The soft gold fillings are also illy adapted for masticating surfaces. There is little or no cohesion in the mass, and therefore, when exposed to the force of mastication, they disintegrate and come out piecemeal.

If the filling, when completed, is to reach a coronal surface, the very soft or non-cohesive foil should not be chosen. You desire a hard surface and a homogeneous mass, and these qualities in a filling are only secured by gold that is more or less adhesive. But the most of our work, even though a part of it be coronal, extends to a proximal, or buccal, or lingual surface, where perfect adaptation is the first requisite. A little consideration, then, will teach you that such fillings should be commenced, and, indeed, mainly constructed of gold that has very little of adhesiveness, this to be changed for that which will produce a hard, coherent mass, when the crown or contoured portion of the work is approached. The most of our manufacturers now make foil that, in its natural state, is soft, but which when submitted to the flame of the annealing lamp becomes harder, harsher, and more adhesive, and these kinds of foil are those most used by good operators.

What, then, may be deduced from these facts? It is that the judicious dentist will so select his foil that he will have just the kind that is best adapted to the case in hand. He will not follow a rigid, unbending rule, making it his boast that he uses only cohesive or non-cohesive gold, but he will employ anything, everything that will best serve to further the end he has in view—the permanent conservation of the dental organs, with a view to their greatest usefulness.

